# Measuring standing hindfoot alignment: reliability of different approaches in conventional x-ray and cone-beam CT

**DOI:** 10.1007/s00402-021-03904-1

**Published:** 2021-04-22

**Authors:** Leonard Simon Brandenburg, Markus Siegel, Jakob Neubauer, Johanna Merz, Gerrit Bode, Jan Kühle

**Affiliations:** 1 Department of Oral and Maxillofacial Surgery, Albert-Ludwigs University Freiburg, Hugstetterstr. 55, 79106 Freiburg, Germany; 2 Department of Orthopedics and Trauma Surgery, Albert-Ludwigs University Freiburg, Hugstetterstr. 55, 79106 Freiburg, Germany; 3 Department of Radiology, Medical Center-University of Freiburg, Faculty of Medicine, University of Freiburg, Hugstetterstr. 55, 79106 Freiburg, Germany; 4 Sporthopaedicum Straubing, Bahnhofsplatz 27, 94315 Straubing, Germany

**Keywords:** Hindfoot alignment, Cone-beam CT, Ankle joint, Malalignment, Saltzman View

## Abstract

**Introduction:**

Currently there is no consensus how hindfoot alignment (HA) should be assessed in CBCT scans. The aim of this study is to investigate how the reliability is affected by the anatomical structures chosen for the measurement.

**Materials and methods:**

Datasets consisting of a Saltzman View (SV) and a CBCT of the same foot were acquired prospectively and independently assessed by five raters regarding HA. In SVs the HA was estimated as follows: transversal shift between tibial shaft axis and heel contact point (1); angle between tibial shaft axis and a tangent at the medial (2) or lateral (3) calcaneal wall. In CBCT the HA was estimated as follows: transversal shift between the centre of the talus and the heel contact point (4); angle between a perpendicular line and a tangent at the medial (5) or lateral (6) calcaneal wall; angle between the distal tibial surface and a tangent at the medial calcaneal wall (7). Intraclass correlation coefficients (ICC) were calculated to assess inter-rater reliability. A linear regression was performed to compare the different measurement regarding their correlation.

**Results:**

32 patients were included in the study. The ICCs for the measurements 1–7 were as follows: (1) 0.924 [95% CI 0.876–0.959] (2) 0.533 [95% CI 0.377–0.692], (3) 0.553 [95% CI 0.399–0.708], (4) 0.930 [95% CI 0.866–0.962], (5) 0.00 [95% CI − 0.111 to 0.096], (6) 0.00 [95% CI − 0.103 to 0.111], (7) 0.152 [95% CI 0.027–0.330]. A linear regression between measurement 1 and 4 showed a correlation of 0.272 (*p* = 0.036).

**Conclusions:**

It could be shown that reliability of measuring HA depends on the investigated anatomical structure. Placing a tangent along the calcaneus (2, 3, 5, 6, 7) was shown to be unreliable, whereas determining the weight-bearing heel point (1, 4) appeared to be a reliable approach. The correlation of the measurement workflows is significant (*p* = 0.036), but too weak (0.272) to be used clinically.

## Introduction

Hindfoot malalignment is crucial for the development of foot and ankle disorders [1]. Pathological pressure peaks due to hindfoot malalignment can lead to degenerative damage, resulting in permanent pain and decreased mobility [2]. Knowledge of the underlying pathology is elementary to surgical therapy for the ankle. Early detection of hindfoot malalignment and adequate treatment therefore plays an important role in the management of patients with foot pain [1, 3].

The most common method in evaluating hindfoot alignment is conventional radiography [4]. Several recording techniques have been established: The Saltzman View (SV), the Long Axial View and the Méary’s View are valuable alternatives [5]. Each of these methods use different anatomical structures as reference, as there is no consensus about the superiority of a method over one other [5].

Even if conventional radiography is a widely used and well-established diagnostic method, conceptual shortcomings are frequently pointed out in literature: superposition effects, inaccuracies in orienting the patient inside the x-ray device and the missing third dimension lower the diagnostic value of this imaging modality [6, 7]. A detailed investigation of single bone structures does not seem to be applicable for conventional radiography [2, 8, 9]. Moreover, the correlation of conventional radiography and clinical findings is questionable [10].

Modern cone-beam CTs (CBCT) can also be used for obtaining imaging of the foot and ankle in a physiological standing position [11]. CBCT imaging has been shown to be more precise in measuring bone position than conventional radiography [4]. The three-dimensional dataset provides additional information about the complex anatomy of foot and ankle, and uses lower radiation dosages than comparable multi-slice CT scans [12, 13]. Despite the large amount of information used in this 3D-imaging modality, conventional radiographs are still routinely taken out to investigate hindfoot alignment [14]. The greater complexity of cross-sectional imaging complicates the evaluation of the dataset. Some proposals for using CBCT for measuring hindfoot alignment have already been published [4, 6, 15–18]. Most of them use 3D models as a basis for evaluation, accompanied by special data preparation and requiring special software tools. This could cause additional costs and should be considered when a cost-effective diagnostic workflow is desired.

As there is no consensus on the anatomical structures which should be taken into account when assessing HA properly in WB-CBCT and x-ray imaging, this study investigates if the reliability of different measurement methods is dependent on the anatomical structures chosen for the measurement method. Unlike recent studies, elaborate volume rendering and the generation of 3D surface models were omitted, developing a simple and less costly measurement method which can be performed using conventional picture archiving and communication systems.

## Materials and methods

In a prospective consecutive study standard digital radiographs in a physiological standing position and weight-bearing cone-beam computer tomographs (WB-CBCT) of the foot and ankle were included starting February 1st, 2016 and ending at January 31st, 2019.

### Inclusion and exclusion criteria

The inclusion criteria were presentation at the outpatient clinic of our department, minimum age of 18 years, the ability to bear their weight fully on both feet and the indication for a SV and a WB-CBCT of the same foot. Patients were included regardless of underlying pathology or existence of radio-opaque implants. The field of view (FOV) of the WB-CBCT had to be large enough to depict the whole foot.

The exclusion criteria were age under 18 years, no indication for SV and WB-CBCT or inadequate image quality, such as motion artefacts, wrong positioning of the patient in the x-ray device or incomplete depiction of the foot.

The study was reviewed and conducted in accordance with the ethical standards of our institution. The study protocol was approved (No. 563/18) by the ethics committee of the […] and obtained written consent from all participants.

### Image acquisition

The indication for standard radiography and WB-CBCT was defined following the local standard. All patients presenting pain symptoms of the foot and ankle received a SV for investigation of the HA. The indication for additional WB-CBCT was only given when single bone structures or their internal structure had to be examined and existing radiographs turned out to be insufficient for this purpose. If possible, only part of the foot was imaged to keep the radiation dose as low as possible.

The SVs were acquired in a physiological standing position using different digital standard radiograph devices of our department. The second ray of the foot was oriented parallel to the central x-ray beam. The detector panel was aligned perpendicular to the central x-ray beam as described by Cobey [9].

The WB-CBCT scans were performed using the Planmed Verity extremity scanner (Planmed Oy, Helsinki, Finland, slice thickness of 0.2 mm, FOV 13 × 16 × 16 cm). The patient had to stand one-legged inside the gantry and placed the contralateral knee on a platform.

SVs and WB-CBCTs were exported to the local picture archiving and communication system (PACS), which is available by default for all physicians at the university medical department (Agfa HealthCare IMPAX EE R20 XVII SU4, Mortsel, Belgium).

### Radiological evaluation

The seven different measurements were performed using conventional PACS of our department. Unlike other recent publications [11, 14, 15], this study omitted volume rendering of the CBCT dataset to enable a comparatively fast and simple examination of the CBCT dataset, as with conventional radiography.

#### Observers

Five raters from the […] performed a step-by-step evaluation of the SVs and WB-CBCTs using an illustrated instruction guide. Two of the raters were physicians at the department for radiology, two raters were surgeons at the department for traumatology and orthopaedics, and the fifth rater was a medical student. X-rays were reviewed, blinded to CBCT measurements, and made on separate occasions. The heterogeneity of the observer group was chosen to avoid possible bias depending on the educational background of the observer.

#### Measurement protocols for evaluation of SV

**Fig. 1:**
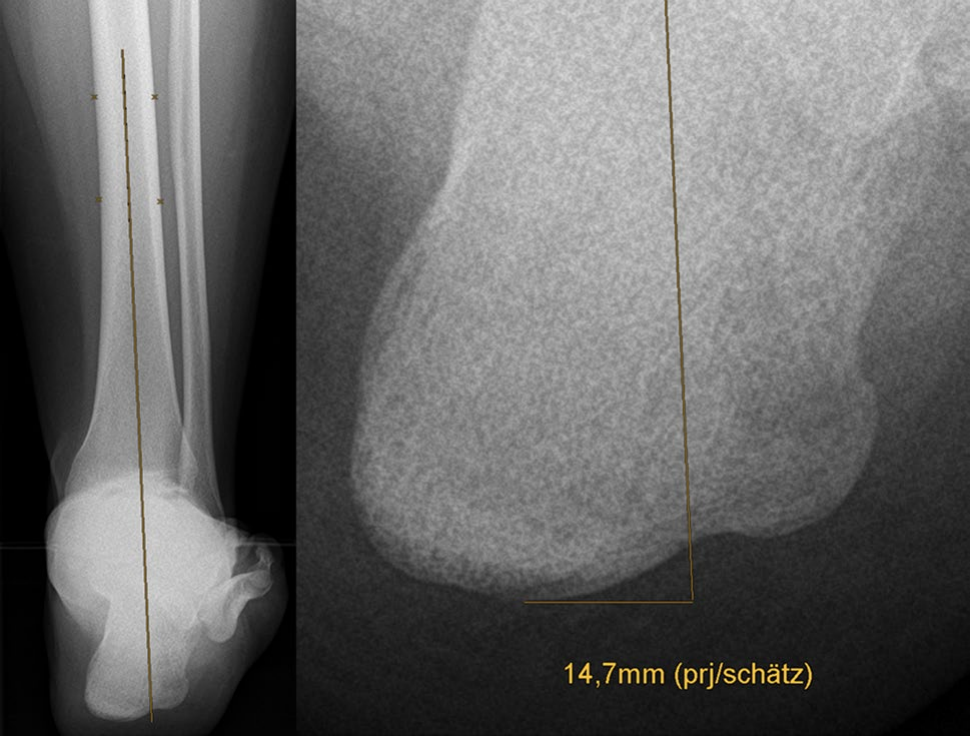
Measurement method 1, in accordance with Saltzman and El-Khoury. Left: Determination of the weight-bearing axis of the tibial shaft. Right: Measuring the transversal shift between the axis of the tibial shaft and the contact point of the heel

**Fig. 2:**
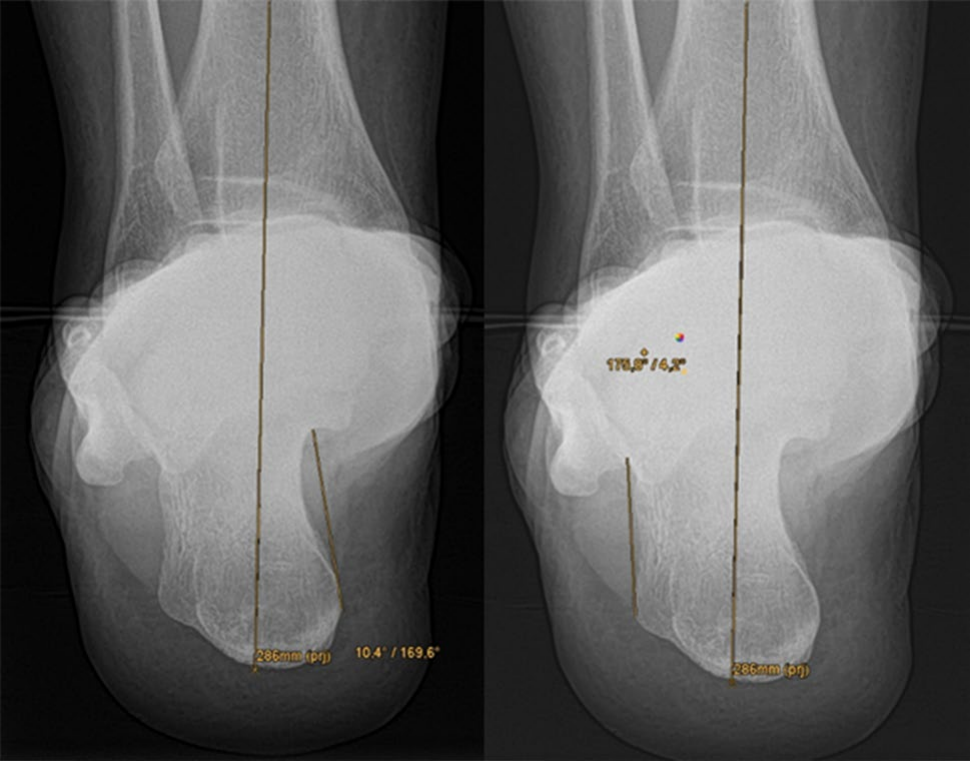
Left: Measurement method 2 determines an angle between the tibial shaft axis and a tangent at the medial contour of the calcaneus. Right: Analogue measurement technique 3 for the lateral calcaneal wall

1. In measurement method one the HA was determined as suggested by Saltzman and El-Khoury [19]. In this procedure, the moment arm of the ankle was estimated by measuring the transversal shift between the weight-bearing axis of the tibial shaft and the contact point of the heel in the SV (Fig. 1). The measurement result was given in millimetres.
2. Measurement method two determines an angle between the tibial shaft axis and a tangent line at the medial contour of the calcaneus. The tangent was defined by two points: the point of the tuber calcanei that protrudes furthest medially, and the point of the sustentaculum tali where the curvature is strongest (Fig. 2). The measurement result was given in degrees.
3. Measurement method three was performed analogously to measurement method two. Instead of the tangent line at the medial wall of the calcaneus, another tangent was set at the two most lateral elevations of the lateral calcaneus wall (Fig. 2). The result of the measurement was also given in degrees.

#### Measurement protocols for evaluation of CBCT

To ensure consistent spatial orientation, the WB-CBCT datasets were first oriented. For this purpose, the border between the caput tali and the trochlea tali (at the beginning of the cartilaginous articular surface) was determined in the sagittal view. Subsequently in the axial view the anterior and posterior border of the talar dome were oriented horizontally.

**Fig. 3:**
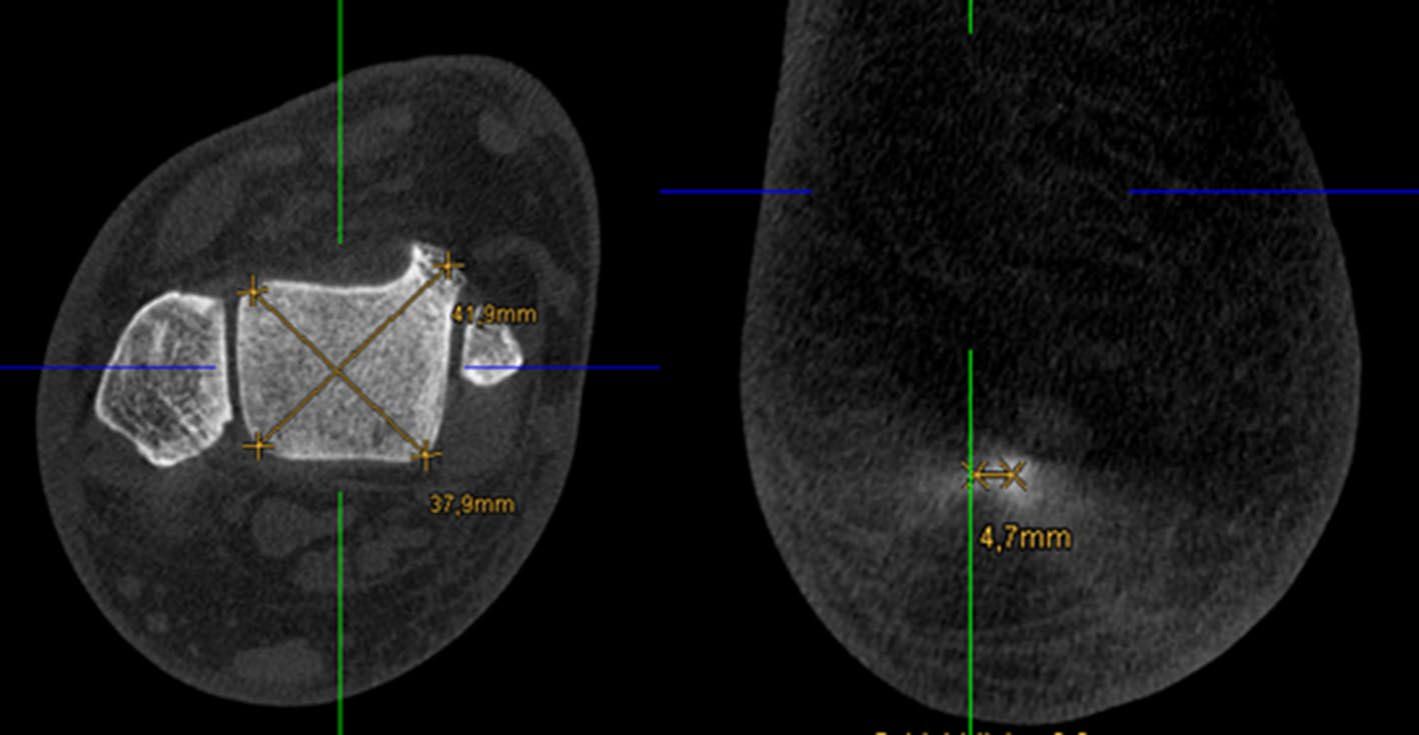
Left: Determination of the centre of the talar dome in the axial view, using two diagonal lines. Right: Projecting of the centre of the talar dome (represented by the green line) perpendicular to the ground and determination of the transversal shift

**Fig. 4:**
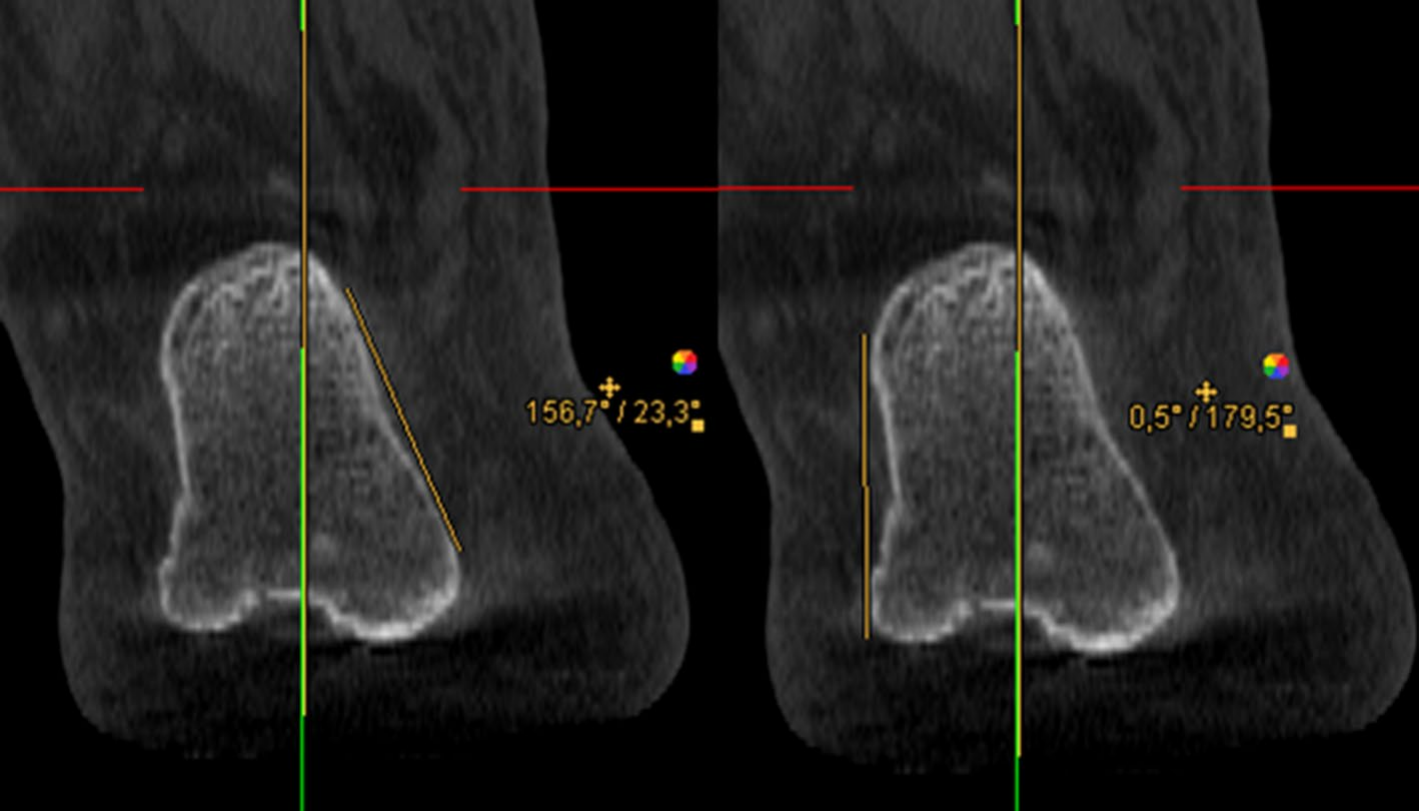
Left: Measurement method 5 determines an angle between a perpendicular line and a tangent line at the contour of the medial calcaneus. Right: Analogous procedure for measurement 6 using the lateral border of the calcaneus

**Fig. 5:**
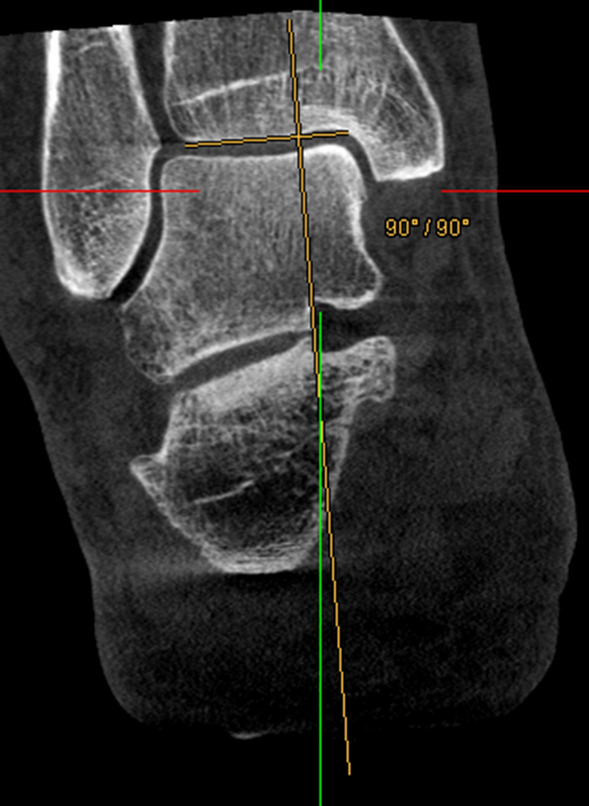
Measurement method 7. Angle between a line perpendicular to the distal tibial articular surface (yellow) and a vertical line (green)

4. In measurement method four the translation of the contact heel point to the centre of the talus was measured. Analogously to the workflow of Saltzman and El-Khoury, the translation of the contact heel point to the weight-bearing centre of the joint was determined with this measurement in the CBCT dataset. As the FOV of the CBCT scans did not show a proper section of the tibia, the centre point of the talus was determined instead of the tibial shaft axis (Fig. 4), and subsequently projected perpendicularly towards the most plantar slice of the dataset, representing the contact point of the heel (Fig. 3). The transversal shift between the two points was given in millimetres.
5. In measurement method five an angle between a perpendicular line and a tangent line at the contour of the medial calcaneus was determined. The contour of the calcaneus was depicted at the level of the most posterior point of the subtalar joint in the coronal view. The angle between a perpendicular line and a tangent line at the medial wall of the calcaneus was measured (Fig. 4). The result of the measurement was given in degrees.
6. In measurement method six an angle between a perpendicular line and a tangent line at the contour of the lateral calcaneus was determined. The procedure was the same as in measurement five, except that the lateral wall of the calcaneus was considered (Fig. 4). The result of the measurement was given in degrees.
7. In measurement method seven an angle between the medial calcaneus wall and a line perpendicular to the distal tibial articular surface, in accordance with Hirschmann et al. [9] was determined. Two lines were defined for angle measurement in coronal view. The first was perpendicular to the distal tibial joint surface at the centre of the tibia, and the second was chosen tangentially along the medial cortex of the calcaneal wall at the level of the tibia’s posterior edge (Fig. 5). The result of the measurement was given in degrees.

All the results had to specify whether a medialization or lateralisation of the contact heel point showed a valgus or varus malalignment of the ankle [1].

### Statistics

The results were documented by each rater using a pre-formed Microsoft Excel worksheet (Microsoft Corporation, Microsoft Excel^®^ Version 16.0). Measured values that were not assessable were counted as missing values in the statistical analysis.

Statistical analyses were performed using SPSS version 21 [19].

For the assessment of the inter-rater reliability, the intra-class correlations were determined. A two-way mixed model with absolute agreement was chosen. A *p*-value of 0.05 was used for outlining statistical significance. Based on the 95% confidence interval of the ICC estimate according to Koo and Li [20], ICC values less than 0.5 are indicative of poor, 0.5 to 0.75 for moderate, 0.75 to 0.9 for good, and greater than 0.90 for excellent reliability.

For a comparison of the two most reliable workflows (one of each modality), a linear regression was performed subsequently. To illustrate the comparability of the measurements a Bland–Altman Plot was made.

## Results

A total of 96 patients received both imaging modalities during the data collection period. 56 cases were excluded because they did not meet imaging requirements (50 because the hindfoot was not entirely depicted in the CBCT dataset, four because of wrong positioning inside the x-ray device, two because of motion artefacts). Further eight patients were excluded because they did not agree to participate in the study.

Eventually 32 patients underwent data evaluation. Twenty men and 12 women were included. Fourteen left and 18 right feet were investigated. The mean age of the patients was 49.4 ± 13.4 years.

1120 measurements were performed by the raters in total and documented in the worksheet supplied. 96 measurements were designated as not evaluable and treated as missing value.

The ICCs for the measurements 1–7 were as follows: (1) 0.924 [95% CI 0.876–0.959] (2) 0.533 [95% CI 0.377–0.692], (3) 0.553 [95% CI 0.399–0.708], (4) 0.930 [95% CI 0.866–0.962], (5) 0.00 [95% CI − 0.111 to 0.096], (6) 0.00 [95% CI − 0.103 to 0.111), (7) 0.152 [95% CI 0.027–0.330]. Table 1 shows the ICCs for all the measurements (conventional radiography and CBCT).

**Table 1:** ICC values for inter-rater reliability for measurement methods 1–7

Measurement	ICC	95%—confidence interval
Measurement 1	0.924	0.876–0.959
Measurement 2	0.533	0.377–0.692
Measurement 3	0.553	0.399–0.708
Measurement 4	0.930	0.866–0.962
Measurement 5	0.00	(−) 0.111–0.096
Measurement 6	0.00	(−) 0.103–0.111
Measurement 7	0.152	0.027–0.330

A linear regression between measurement 1 and 4 showed a correlation of 0.272 (*p* = 0.036).

The Bland–Altman Plot shows that measurement method 4 tends to measure smaller amounts of translations in comparison to measurement method 1.

## Discussion

### Main findings

The main finding of this study was that the reliability of measurement methods regarding HA vary remarkably depending on the chosen anatomical structure.

Only measurement methods estimating the hindfoot moment arm by determining the contact heel point appeared to be reliable in this study. The evaluation of the SV with measurement method one, as described by Saltzman and El-Khoury [21], turned out to be reliable, with an ICC of 0.924 [95% CI 0.876–0.959]. Method four for CBCT datasets was comparably reliable with an ICC of 0.930 [95% CI 0.866–0.962]. According to Koo and Li [20] both measurement methods achieved excellent inter-rater reliability, which is crucial for developing a valid and clinically usable measurement method.

Measurement methods which have the calcaneal contour as a reference to assess HA are less reliable. In a conventional x-ray, this applies to measurement methods two and three, which showed only moderate reliability with ICCs of 0.533 (method two) and 0.553 (method three) [20]. In the CBCT dataset, the calcaneal contour was considered in measurement methods five, six and seven, which had poor reliability (method five: 0.0 [95% CI − 0.111 to 0.096], method six: 0.0 [95% CI − 0.103 to 0.111], method seven: 0.152 [95% CI 0.027–0.330]) [20]. Even the availability of a detailed and illustrated step-by-step instruction guide for measurement performance could not lead to a more uniform evaluation of the datasets when these measurement methods were used.

### Limitations

The simple design of conventional x-ray units enables a fast and viable adjustment of the FOV. The lower radiation doses applied in conventional radiography may justify the use of a wide FOV, as the cumulative radiation exposure of this examination is known to be comparably low [22, 23]. This guarantees the adequate depiction of a long section of the tibia and an intuitive and feasible way to determine the shaft axis of the tibia, which is a widely used way to measure HA [6, 9, 11, 15, 15, 18, 21].

In contrast the small FOV (16 × 16 × 13) of the WB-CBCT scanners used barely depicts the tibia. Thus, another anatomical structure than the tibia shaft axis had to be chosen determining HA in WB-CBCT. Method four takes the load bearing centre of the talus as alternative proximal reference structure (Fig. 3). The anatomical axis of the leg contains the tibial shaft axis and is supposed to cross the centre of the talus and the contact heel point [14, 24]. A deviation of the centre of the talus and the contact heel point may therefore indicate a malalignment of the ankle. It is important to note that the validity of this approach was not proven and remains questionable. There is a possibility for deviation of the two measurements in cases with varus or valgus angulation, as the tibial shaft axis does not cross the centre of the tibia in these cases anymore.

This study included all consenting patients who received a WB-CBCT and a conventional x-ray of the hindfoot between February 2016 and January 2019 without further pre-selection. Thus, a heterogeneous study population was acquired with different diagnoses and different HA. Also, some patients had radio-opaque implants, and some did not. This must be considered when interpreting the results, as the effect of these features on the reliability of the measurements is unknown. Due to a restricted indication for double imaging (SV and WB-CBCT) only a small number of individuals could be included in this study, which made the evaluation of subgroups impossible. This describes another limitation of the study, which should be overcome in future studies by enlarging the study population.

### Reliability of measurements using the contact heel point (metric measurements)

The measurement methods one and four estimate the hindfoot moment arm by measuring horizontal translation between the tibial shaft axis, respectively, the centre of the talus and the contact heel point.

The reliability of measurement method one was already determined by its developers Saltzman and El-Khoury [21] and yielded an inter observer coefficient of 0.97. In our study the excellent reliability of this measurement method could be confirmed (ICC = 0.924 [95% CI 0.876–0.959]). Determining tibial shaft axis and extending it downwards for measuring the horizontal distance to the heel contact point showed to be a reliable measurement. Angular measuring techniques using the SV have been investigated previously and showed remarkably poorer reliability [1, 25]. Measuring horizontal translation instead of angles seems to be more reproducible and can be recommended when excellent reliability is required, e.g. when a case is assessed jointly by two physicians.

In measurement method four the authors tried to imitate the approach of the measurement method one in the cross-sectional CBCT dataset. Because the determination of the tibial shaft axis was not possible in our datasets, the centre of the talus was used as proximal reference structure instead. To our knowledge this approach for estimating HA was not published before.

The following features leave scope for interpretation and could have affected the reliability of measurement method four: (1) Choosing different axial image lines could affect the determined position of the centre of the talus. (2) The contact heel point had to be selected in the most plantar slice, which showed a circular bone-area rather than a real point (Fig. 4). Surprisingly, despite these issues the measurement method four has shown to be reliable with an ICC of 0.930 [95% CI 0.866–0.962].

### Reliability of measurements using the calcaneal contour (angular measurements)

For measurement methods two and three a tangent at the medial respectively lateral contour of the calcaneus is created and the angle enclosed by the tangent and the tibial shaft axis is determined. Both measurements acquire moderate reliability (0.533 [95% CI 0.377–0.692], respectively 0.553 [95% CI 0.399–0.708]).

A review of the measurements performed within the SV showed that placing a tangent line at the lateral or medial contour of the calcaneus remains a margin for interpretation. The complex morphology of the sustentaculum tali, as well as bone prominences and osteophytes at the lateral and medial wall, yielded varying results and poor to moderate reliability.

Dagneaux et al. [25] investigated different angular measurement techniques using the SV leading to ICCs between 0.22 and 0.66. Reilingh et al. [1] compared angular measurement techniques using different x-ray techniques. Poor to moderate inter-rater reliability could be achieved for angular measurements using the SV (ICC of 0.49 in unilateral and 0.58 in bilateral stand). Even if the authors didn’t use the exact same way to measure HA-angles, excellent reliability couldn’t be achieved likewise. Thus the use of angular measurement techniques in conventional radiography has been proofed to be accompanied by observer-dependent and variant results in this as well as in previous studies.

Measurement techniques five, six and seven equally placed a tangent line at the calcaneal contour to perform angular measurements.

It could be assumed that the use of cross-sectional images might lead to higher reliability, as superposition effects are absent, and the calcaneal contour could therefore be evaluated more consistently. Surprisingly, methods 5, 6 and 7 in the CBCT dataset turned out to be even less reliable. Further investigation shows that despite the detailed step-by-step instructions for the measurements, the raters navigated in different coronal image lines. Even if adjacent image lines were taken into account, the resulting tangent lines at the calcaneal contour varied remarkably in their spatial orientation. This may explain the poorer reliability of methods 5–7 when compared to 2 and 3.

De Cesar et al. [15] reported an ICC of 0.73 in inter-rater reliability for a measurement procedure, which also takes the contour of the calcaneal wall into account. In this study, the calcaneal axis was determined by bisecting 2 transversals between two lines adapted to the lateral and medial osseous contours of the calcaneus, as previously described by Williams et al. 2014 [14, 15]. The remarkably higher ICC in the study of De Cesar et al. could be explained by the use of 3D models, which forgo the problem of selecting image lines and enable adapting tangent lines over a multitude of cross-sectional image lines in an intuitive and unambiguous manner [6, 11, 15]

Hirschmann et al. previously investigated the reliability of measurement method seven in WB-CBCT [16] and achieved an ICC of 0.83 for inter-rater reliability. In this study, two trained musculoskeletal radiologists performed the measurement and the study population seems to be more consistent (e.g. no patients with endoprosthesis were included). The datasets in our study were evaluated by physicians with different specialisations and different levels of experience. The approach to analysing datasets may vary between clinicians and radiologists, which may explain the high variance and therefore the poorer reliability of our measurement results compared to Hirschmann et al. Moreover, it remains to question if the heterogeneity of our study population further complicated the evaluation and decreased reliability likewise.

Several publications which used three-dimensional metrics for measuring calcaneal angulation found higher reliability for their measurements [11, 14, 15]. The mentioned difficulties with data set alignment and selecting imaging lines are absent when using three-dimensional volumes for measuring HA. This describes a major advantage of using three-dimensional models and may justify a more costly and time-consuming processing of volume rendering and performing special 3D-measurements.

### Correlation of measurement method one and four

**Fig. 6:**
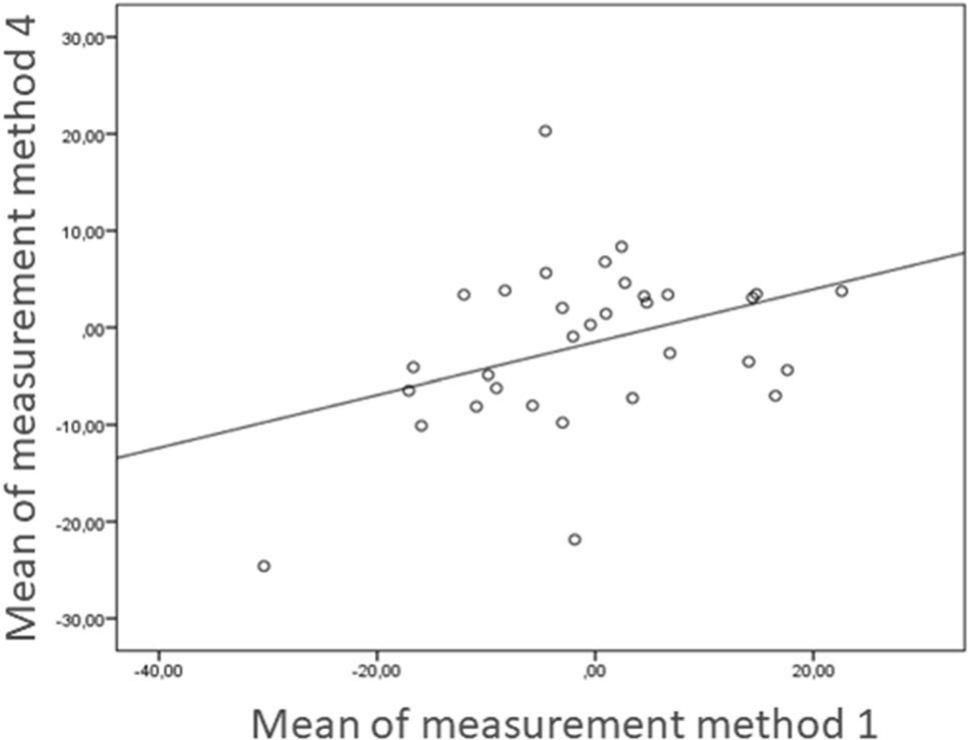
Linear regression of measurement method 4, leading to a positive correlation of 0.272 (*p* = 0.036)

**Fig. 7:**
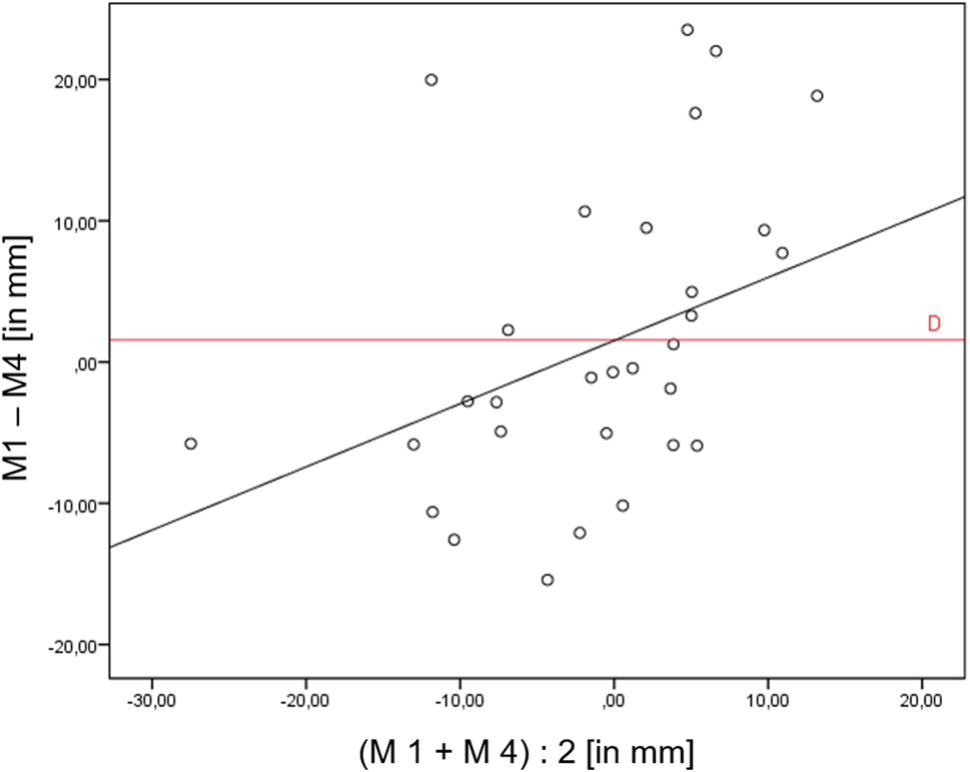
Bland–Altman Plot for comparison of the two most reliable measurement methods in SV (method 1) and WB-CBCT (method 4). On the *X*-axis the means of both measurement methods are plotted. On the *Y*-axis the difference of measurement method 1 and 4 is plotted. The distribution of the measurement points indicates that for mean values > 0 (*x* > 0) there is mostly a positive difference (*y* > 0). However, the differences at mean values < 0 (*x* < 0) tend to result in a negative difference. Therefore, it can be concluded that measurement method four estimates the severity of the deformity to be lower than measurement method one

To compare the most reliable measurement methods of the SV and the WB-CBCT, a linear regression of the measurement methods one and four was performed. The low correlation of 0.272 (*p* = 0.036, Fig. 6) indicates that the two methods, although reliable, measure different things. Measurement method four measures the translation of the tibial shaft axis to the heel contact point and measurement method 1 determines the translation of the centre of the talus to the heel contact point. The Bland–Altman Plot in Fig. 7 plots the difference of the two measurements (*Y*-axis) against the arithmetic mean of the two measurements (*X*-axis). The resulting graph facilitates the assessment of the agreement between the two measurements, as well as the identification of value ranges with particularly good or poor agreement. It can also draw attention to the fact that one method overestimates high values and underestimates low values.

In our study measurement method four estimates the severity of the deformity to be lower than measurement method one (Fig. 7). This may be explained, because the talus centre does shift in only a small extent, whereas angulation of the tibial shaft axis changes more remarkably in case of hindfoot misalignment. Thus, even if both approaches could possibly in some way detect a misalignmet of the hindfoot, the two measurement methods cannot be compared directly. To increase the correlation of measurement methods one and four it would be helpful to use the same proximal reference structure in WB-CBCT and x-rays (e.g. tibial shaft axis). Burssens et al. [6] have already shown that a short distal tibial section can be sufficient for determining a valid tibial shaft axis. Therefore, CBCT scanners with a little larger FOV should be used in further studies, so that a short tibial section can be depicted, and the tibial shaft axis can be determined equally in the CBCT dataset. Implementing the tibial shaft axis in measuring method four instead of the centre of the talus would therefore raise the comparability of methods one and four. This could strengthen the correlation of the two methods, and may improve the diagnostic value of the novel measurement method four. Moreover, a comparison of both measurement methods with clinical measuring procedures should be performed to check for validity.
